# The risk between thyrotropin suppression and bone mineral density in differentiated thyroid cancer

**DOI:** 10.1097/MD.0000000000031991

**Published:** 2022-12-02

**Authors:** Yang Zou, Bin Li, Xiaodong Wang, Jingxin Mao, Yanyan Zhang

**Affiliations:** a Department of Endocrinology, Jiangjin Center Hospital (Chongqing University Jiangjin Hospital), Chongqing, China; b Department of Pharmacology, Chongqing Medical and Pharmaceutical College, Chongqing, China; c College of Pharmaceutical Sciences, Southwest University, Chongqing, China.

**Keywords:** bone mineral density, differentiated thyroid cancer, meta-analysis, risk factor, thyrotropin suppression

## Abstract

The effect of thyroid stimulating endocrine (TSH) suppression medical aid on bone mineral density (BMD) of patients with differentiated thyroid carcinoma (DTC) or differentiated thyroid malignant neoplastic disease is still controversial. Our aim was to investigate the effect of TSH suppression therapy on BMD of patients with DTC.

**Methods:**

A total of 1651 DTC patients with TSH-suppression medical care were analyzed by RevMan 5.3 software (https://training.cochrane.org/online-learning/core-software/revman/revman-5-download) in the present study. The PubMed and Embase databases were consistently hunted for works revealed through July 29, 2022.

**Results:**

The results indicated that a significant association between femoral bone mineral density (FN-BMD) (*P* = .02) or lumbar spine bone mineral density (L-BMD) (*P* = .04) and DTC patients with TSH-suppression therapy. However, the total hip bone mineral density (TH-BMD) was not significantly related to DTC patients with TSH-suppression therapy (*P* = .11). For premenopausal women, it was shown that TH-BMD (*P* = .02) or L-BMD (*P* = .01) were closely related to DTC patients with TSH-suppression therapy. However, there was no relationship between FN-BMD and DTC patients with TSH-suppression therapy (*P* = .06). For postmenopausal women, TH-BMD was closely related to DTC patients with TSH-suppression therapy (*P* = .02). It was revealed that there was no significant difference between L-BMD (*P* = .16) or FN-BMD (*P* = .26) and DTC patients with TSH-suppression therapy. For men, there was no relationship between FN-BMD (*P* = .94) or L-BMD (*P* = .29) and DTC patients with TSH-suppression therapy.

**Conclusion:**

Our systematic review has demonstrated that TSH inhibition treatment mainly influence the TH-BMD or L-BMD of the DTC patients who were premenopausal women; the TH-BMD of the DTC patients who were postmenopausal women. In addition, there was no influence on the FN-BMD or L-BMD of the DTC patients who were men.

## 1. Introduction

Thyroid cancer is a kind of malignant tumor which mainly originates from thyroid follicular cells and parafollicular cells. The pathological classification includes 4 types: Medullary thyroid carcinoma; Follicular thyroid carcinoma; Anaplastic thyroid carcinoma; Papillary thyroid carcinoma. follicular thyroid cancer and papillary thyroid cancer are also called differentiated thyroid carcinoma (DTC).^[1]^ With ultrasound technology, the detection rate of DTC has considerably improved in recent years. Nowadays, the treatment methods on DTC mainly including surgery treatment (underwent thyroidectomy), followed by radioiodine ablation treatment and thyroid stimulating hormone (TSH) inhibition treatment (supraphysiologic doses of L-thyroxine).^[2]^ The progress of DTC is usually slow, and the prognosis is good in most patients with more than a 90% survival rate 10 years after treatment.^[3]^ It was reported that DTC expresses TSH receptor on cell membrane, and TSH stimulates cell growth.^[4]^ The supraphysiological dose of L-thyroxine (LT_4_) to inhibit TSH is a common method of treating DTC, and its purpose is to reduce the risk of relapse.^[5]^

Thyroid hormone has a physiological stimulation effect on bone remodeling and bone mineralization, and normal thyroid status in children and adolescents is a necessary condition to obtain peak bone mass.^[6]^ Exogenous LT_4_ can maintain the normal intake of thyroxine in patients with DTC after total thyroidectomy. However, it has been demonstrated that when inhibiting the dose of TSH by LT_4_ (serum TSH concentrations less than 0.1 mU/L), patients often suffered from subclinical hyperthyroidism.^[7]^ Furthermore, it was reported that subclinical hyperthyroidism may also increase the risk of cataclasis and cardiovascular disease.^[8]^ It was revealed that there exists a positive relation between TSH and bone mineral density (BMD) or osteocalcin (minimal bone anabolic effect).^[9]^ However, the results of the effect of TSH inhibition therapy on BMD is still controversial, and the reference value of the conclusions is limited. Therefore, this meta-analysis was used to evaluate whether or not the inhibition of endocrine with LT_4_ on DTC patients encompasses a negative result on BMD, so as to produce a basis for clinical application.

## 2. Methods

### 2.1. Search strategy

We searched the PubMed and Embase databases respectively for relevant published articles until July 29, 2022. The following keywords were utilized in the search: “differentiated thyroid carcinoma” OR “DTC” AND “TSH-suppression therapy” OR “thyroxine suppression therapy” OR “levothyroxine therapy” AND “bone mineral density” OR “BMD.” Relevant articles were used to broaden the search scope, and all retrieved studies, reviews and conference abstracts were retrieved by the computer. If multiple published studies described the constant population, we opted to extract solely the foremost complete or recent 1. A total of 3 authors (YZ, BL, and YY-Z) independently completed the choice method and resolved the differences through discussion finally.

### 2.2. Selection criteria

The selection strategy used the following criteria: English language studies; Horizontal studies, cohort studies, prospective control studies, retrospective control studies and case-control studies of TSH inhibition therapy for DTC; The TSH level of patients reached the inhibition target; The time of TSH inhibition treatment needed to be provided.

The exclusion criteria was adapted to exclude the following: Non-English language studies; Reviews, congress abstracts, conferences or conference records, case reports, and letters to editors; Studying period beyond 20 years; Insufficient data (e.g., less than 10 patients in the present research); Glucocorticoids or other drugs used in the research that may affect bone metabolism in patients; Participants who underwent any treatment of osteoporosis or osteopaenia.

### 2.3. Data extraction

Two authors (JXM and XDW) independently extracted the following information from the included articles: first author, country, publication years, study design/style, case number, administration time, covariate adjustment, the doses of LT_4_ and Newcastle-Ottawa quality assessment scale. Any disagreements/opinions were resolved by the third investigator (YY-Z). The Newcastle-Ottawa quality assessment scale was utilized to assess the standard of the study.

## 3. Statistical analysis

RevMan version 5.3 software was used for all statistical analysis (Cochrane Collaboration, Oxford, UK). The mean difference (MD) and the 95% confidence interval (CI) were used to calculate each study’s impact’s size. Except as otherwise noted, statistical significance was defined as a *P* value 0.05. The *Q*-test and the *I*^2^ statistic were also used to measure the heterogeneity. A fixed-effect model was used if *P* > .1 and *I*^2^ < 50%; otherwise, a random-effect model was applied. In addition, Begg’s funnel plots have been used to assess publication bias.

## 4. Results

**Figure 1.**
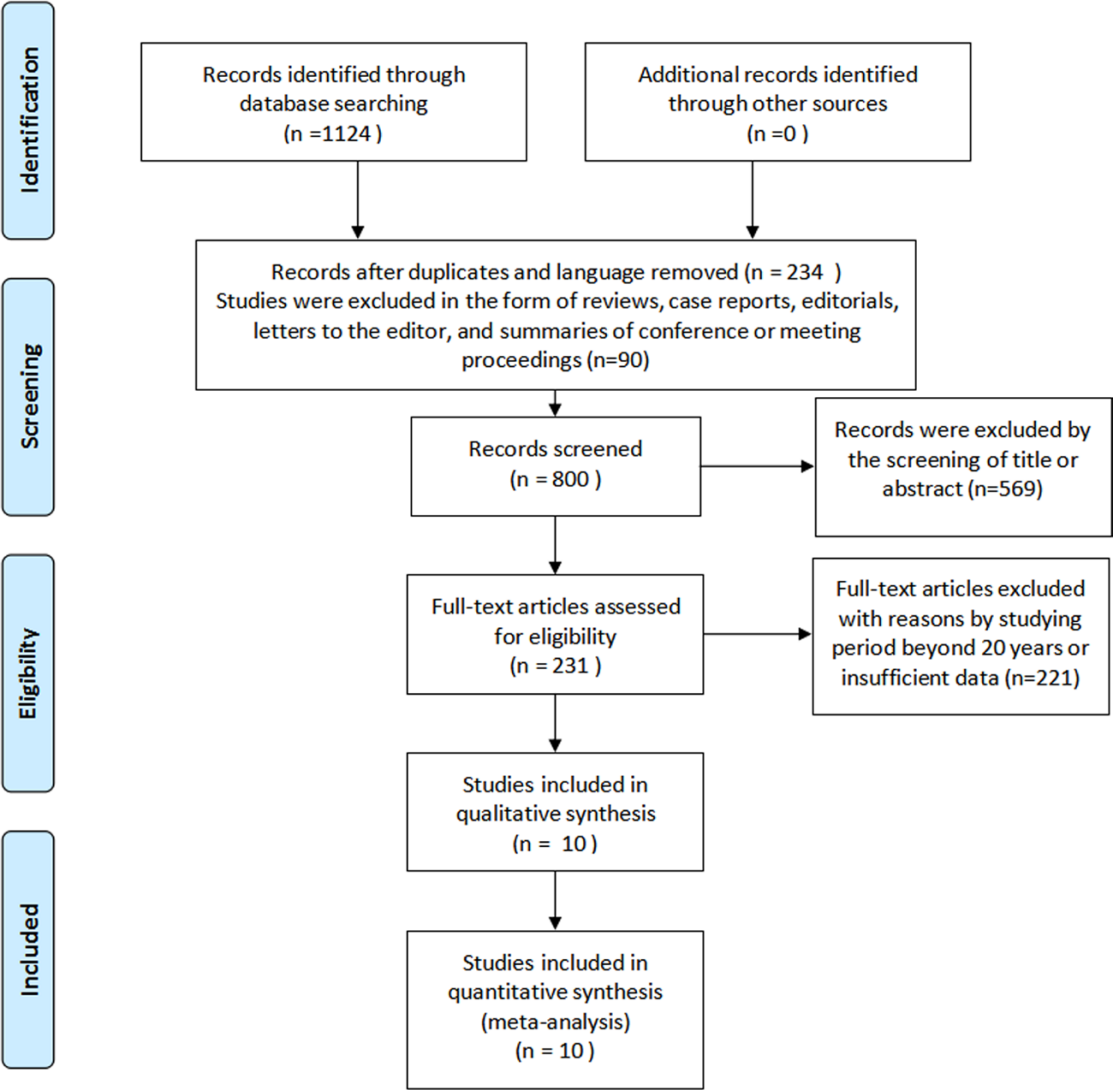
Flow chart of the study selection process (PRISMA flow diagram).

Following the selection process, a total of 1124 studies were at first taken into consideration for the meta-analysis. Among them, 675 studies were from the PubMed database and 449 studies from the Embase database, respectively. A total of 234 records were excluded by language and duplicates, and 90 studies were excluded for being reviews, case reports, editorials, letters to the editor, and summaries of conference or meeting proceedings. A total of 569 records were excluded because of title or abstract screening, and 221 records were excluded due to a study period longer than 20 years or insufficient data (e.g., less than 10 patients in the present research). A total of 10 studies that met our inclusion criteria were ultimately enclosed within the meta-analysis after intensive examination. In general, the literature analyzed in this review has a relatively high quality. Figure 1 displays the analysis choice flow sheet. Table 1 contains the included studies’ basic characteristics.

**Table 1:** Basic characteristics of included studies in the effect of TSH- suppression therapy on BMD in patients with DTC.

First author	Country	Publication yrs	Study design/style	Case number	Administration time (mo)	Covariate adjustment	Doses of LT_4_	NOS
de Barros^[30]^	Japan	2015	Cross-sectional study	17	6–96	Age, BMI, TSH	120 *µ*g/m^2^/kg	6
Dominguez^[31]^	Spain	2018	Prospective study	145	24–264	Age, BMI, TSH, BMD	1.82 ± 0.56 *µ*g/kg	7
Duan^[32]^	China	2020	Prospective study	115	18–108	BMD, FT3, FT4, TSH	25–150 *µ*g/day	8
Eftekhari^[33]^	Iran	2008	Cross-sectional study	66	14–17	BMD, FT3, FT4, TSH	168.33 ± 24.3 µg/day	7
Kim^[34]^	Korea	2015	Prospective study	124	12–18	T4, FT4, TSH, BMD	Following ATA guidelines	7
Moon^[35]^	Korea	2016	Retrospective cohort study	396	36	Age, BMI, TSH, FT4, BMD	150.3 ± 17.2 *µ*g/day	8
Reverter^[36]^	Spain	2005	Cross-sectional study	217	6–12	Age, BMI, TSH, FT4, T4, BMD	195 ± 43 *µ*g/day	7
Schneider^[37]^	Germany	2012	Cross-sectional study	177	6	Age, BMI, TSH, FT4, FT3, BMD	N. A	6
Tournis^[38]^	Greece	2015	Prospective study	169	28	Age, BMI, TSH, FT3, FT4, BMD	139.8 ± 23.9 *µ*g/day	7
Zhang^[39]^	China	2018	Retrospective study	225	6–24	Age, BMI, TSH, FT3, FT4, BMD	Following CTA guidelines	6

ATA = American thyroid association, BMD = bone mineral density CTA = Chinese thyroid association, DTC = differentiated thyroid carcinoma, LT4 = L-thyroxine, NOS = Newcastle-Ottawa quality assessment scale, SH = thyroid stimulating endocrine.

### 4.1. Comparison of levels of TSH between TSH-suppression therapy DTC group and the control group.

The levels of TSH between the tTSH-suppression therapy DTC group and the control group varied in different studies (Table 2). Overall, the *p*-values are significantly different (*P* < .01) between the TSH-suppression therapy DTC group and the control group in this systematic review and meta-analysis study. It was indicated that the TSH-suppression therapy is effective in DTC patients.

**Table 2:** Comparison of levels of TSH between TSH-suppression therapy DTC group and the control group.

References	DTC Patient group TSH (mU/L)	Control group TSH (mU/L)	*P*-value
de Barros	0.16 ± 0.22	1.79 ± 0.96	< .001
Dominguez	0.41 ± 0.57	1.00 ± 1.77	.0046
Duan	0.35 ± 0.14	1.65 ± 1.45	< .001
Kim	0.10 ± 0.07	1.34 ± 0.66	< .001
Moon	0.07 ± 0.09	2.19 ± 1.56	< .001
Reverter	0.02 ± 0.01	2.1 ± 0.9	< .01
Schneider	0.11 ± 0.45	1.86 ± 0	< .001
Tournis	0.14 ± 0.2	1.86 ± 0.9	< .001
Zhang	0.09 ± 0.01	0.63 ± 0.08	< .001

DTC = differentiated thyroid carcinoma, TSH = thyroid stimulating endocrine.

### 4.2. BMD of TSH-suppression therapy group and control group (Table 3, Fig. 2)

A random-effects model was selected, and the inverse variance method was applied to assess the association of TSH-suppression therapy and BMD in DTC patients. The results indicated that a major association between femur neck bone mineral density (FN-BMD) and DTC patients with TSH-suppression medical care (*I*^2^ = 68%, MD = 0.02, 95%CI = 0.01–0.08, *P* = .02, Fig. 3A). It was also demonstrated that the total hip bone mineral density (TH-BMD) was not significantly related to DTC patients with TSH-suppression therapy (*I*^2^ = 65%, MD = 0.04, 95%CI=–0.00–0.09, *P* = .11) (Fig. 3B). In addition, the results showed that the relationship was significant between lumbar spine bone mineral density (L-BMD) and DTC patients with TSH-suppression therapy (*I*^2^ = 78%, MD = 0.05, 95%CI = 0.00–0.11, *P* = .04) (Fig. 3C).

**Table 3:** BMD of TSH-suppression therapy group and control group.

References	Patients	Controls	FN-BMD (g/cm^2^)		TH-BMD (g/cm^2^)		L-BMD (g/cm^2^)	
			Patients	Controls	Patients	Controls	Patients	Controls
de Barros	17	34	1.058 ± 0.17	1.029 ± 0.13	N. A	N. A	1.204 ± 0.14	1.183 ± 0.12
Dominguez	61	84	^*^0.80 ± 0.13	0.67 ± 0.11	0.89 ± 0.11	0.80 ± 0.10	^*^0.99 ± 0.13	0.84 ± 0.15
Eftekhari	22	22	N. A	N. A	N. A	N. A	1.08 ± 0.18	1.05 ± 0.09
Kim	24	24	0.756 ± 0.134	0.747 ± 0.133	0.862 ± 0.148	0.858 ± 0.147	^**^0.993 ± 0.183	0.676 ± 1.340
Moon	17	51	0.930 ± 0.100	0.900 ± 0.090	0.980 ± 0.100	0.960 ± 0.080	1.210 ± 0.110	1.180 ± 0.120
Reverter	44	44	1.032 ± 0.124	1.017 ± 0.125	N. A	N. A	1.229 ± 0.167	1.223 ± 0.155
Schneider	28	29	1.055 ± 0.13	1.015 ± 0.10	1.099 ± 0.14	1.085 ± 0.10	1.253 ± 0.18	1.226 ± 0.13
Tournis	40	29	0.940 ± 0.100	0.900 ± 0.100	0.970 ± 0.100	0.930 ± 0.100	1.200 ± 0.100	1.10 ± 0.100
Zhang	54	71	N. A	N. A	N. A	N. A	1.03 ± 0.15	1.06 ± 0.27

Note: Femoral neck bone mineral density (FN-BMD), total hip bone mineral density (TH-BMD), lumbar spine bone mineral density (L-BMD). The data represent the mean ± SD. ^**^P < .01 or ^*^P < .05 vs control group.

BMD = bone mineral density, FN-BMD = femoral bone mineral density, L-BMD = lumbar spine bone mineral density, TH-BMD = total hip bone mineral density, TSH = thyroid stimulating endocrine.

**Figure 2.**
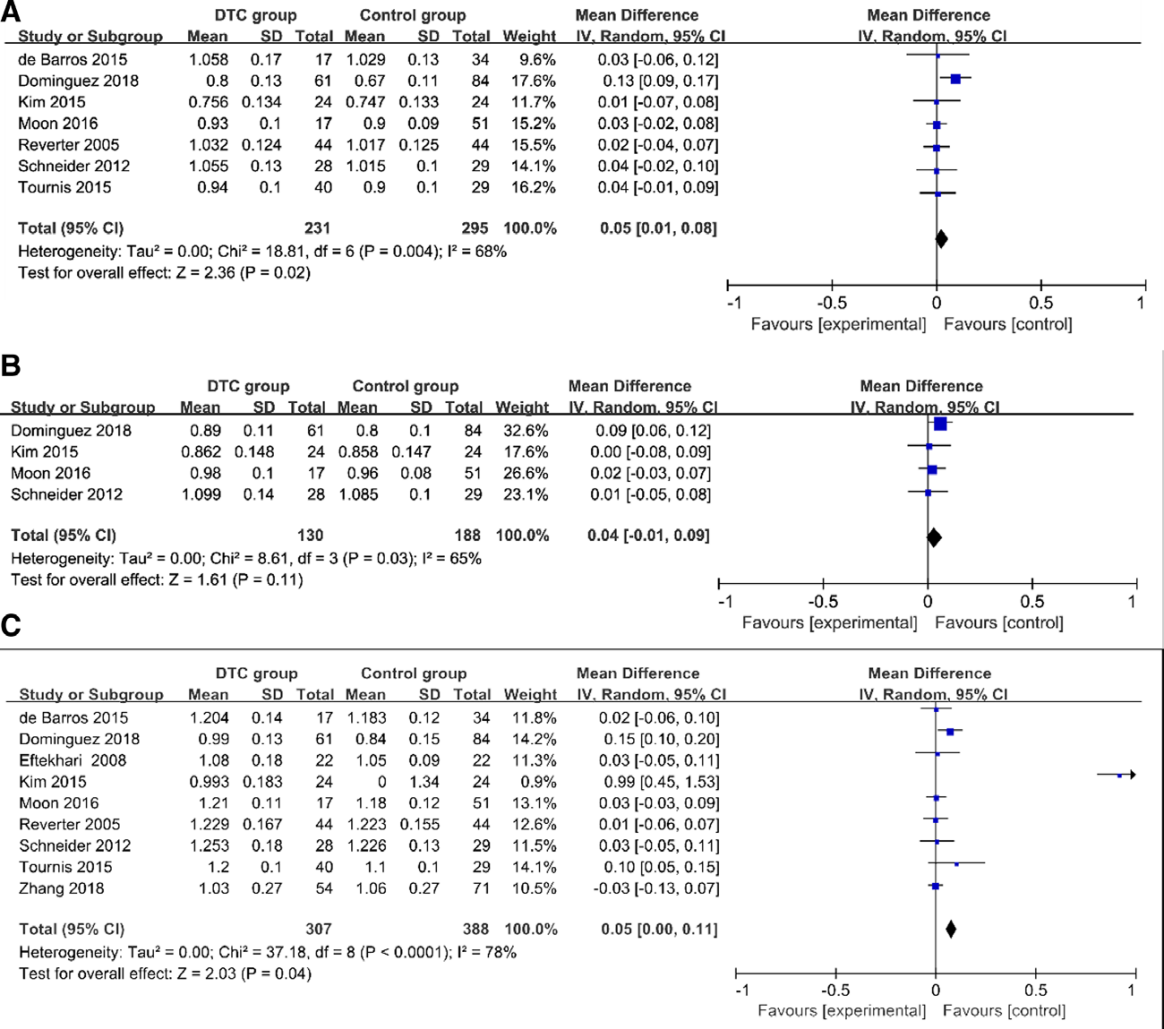
Forest plots of BMD of TSH-suppression therapy group and control group. (A) postmenopausal women; (B) premenopausal women; (C) men. BMD = bone mineral density, TSH = thyroid stimulating endocrine.

**Figure 3.**
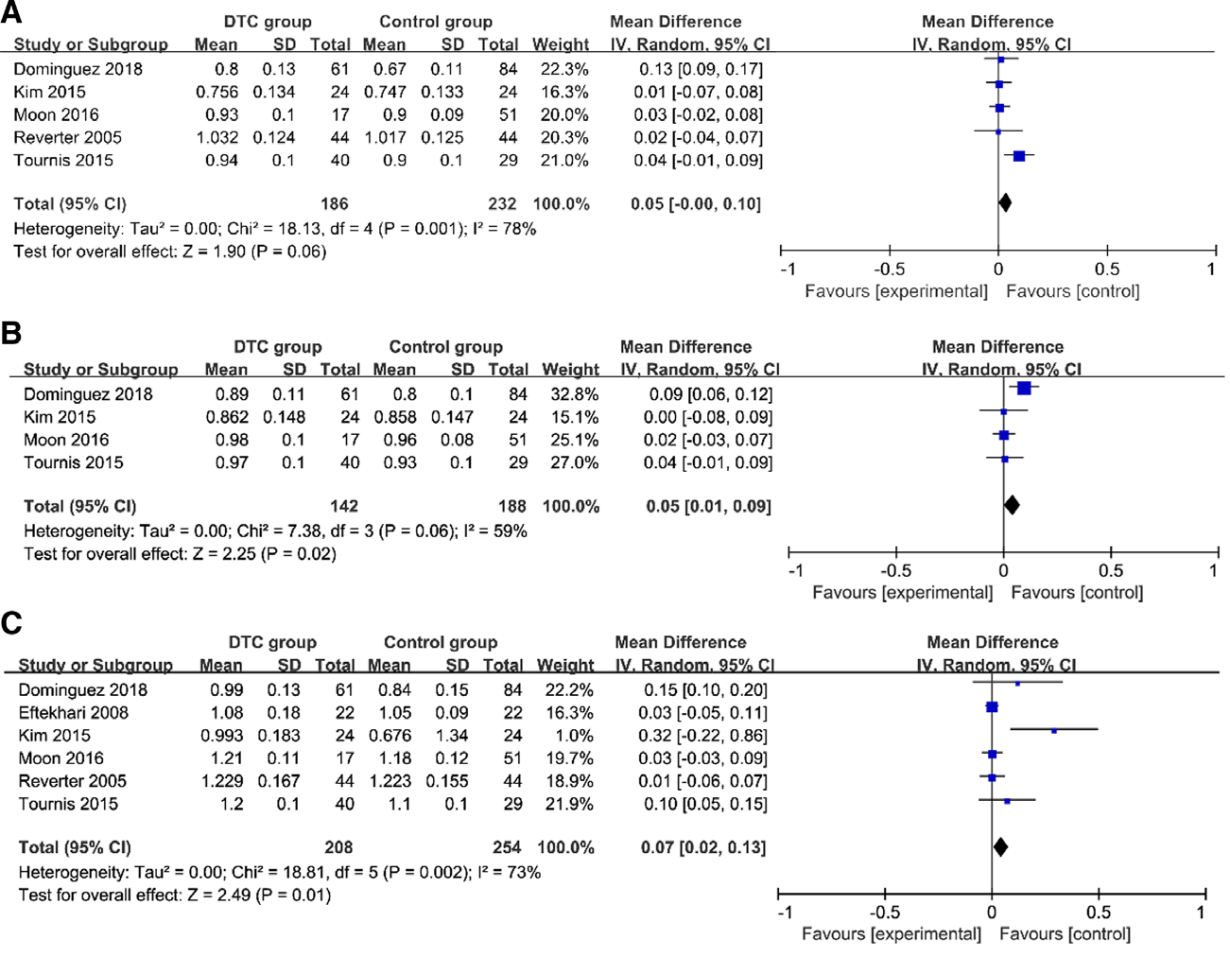
Forest plots of BMD of TSH-suppression therapy group and control group in premenopausal women. (A) FN-BMD; (B) TH-BMD; (C) L-BMD. BMD = bone mineral density, FN-BMD = femoral bone mineral density, L-BMD = lumbar spine bone mineral density, TH-BMD = total hip bone mineral density, TSH = thyroid stimulating endocrine.

### 4.3. BMD of TSH-suppression therapy group and control group in premenopausal women (Table 4, Fig. 3)

A random-effects model was employed, and the inverse variance method was applied to assess the association of TSH-suppression therapy and BMD in premenopausal women with DTC. It was found that there was no relationship between FN-BMD and DTC patients with TSH-suppression therapy who were premenopausal women (*I*^2^ = 78%, MD = 0.05, 95%CI=–0.00–0.10, *P* = .06) (Fig. 3A). However, it was also indicated that TH-BMD was closely related to DTC patients with TSH-suppression therapy who were premenopausal women (*I*^2^ = 59%, MD = 0.05, 95%CI=–0.01–0.09, *P* = .02) (Fig. 3B). It was revealed that there was a significant difference (*P* < .05) in L-BMD and DTC patients with TSH-suppression therapy who were premenopausal women (*I*^2^ = 73%, MD = 0.07, 95%CI=–0.02–0.13, *P* = .01) (Fig. 3C).

**Table 4:** BMD of TSH-suppression therapy group and control group in premenopausal women.

References	Patients	Controls	FN-BMD (gr/cm^2^)		TH-BMD (gr/cm^2^)		L-BMD (gr/cm^2^)	
			Patients	Controls	Patients	Controls	Patients	Controls
Dominguez	61	84	^**^0.80 ± 0.13	0.67 ± 0.11	^*^0.89 ± 0.11	0.80 ± 0.10	^*^0.99 ± 0.13	0.84 ± 0.15
Eftekhari	22	22	N. A	N. A	N. A	N. A	1.08 ± 0.18	1.05 ± 0.09
Kim	24	24	0.756 ± 0.134	0.747 ± 0.133	0.862 ± 0.148	0.858 ± 0.147	^**^0.993 ± 0.183	0.676 ± 1.340
Moon	17	51	0.930 ± 0.100	0.900 ± 0.090	0.980 ± 0.100	0.960 ± 0.080	1.210 ± 0.110	1.180 ± 0.120
Reverter	44	44	1.032 ± 0.124	1.017 ± 0.125	N. A	N. A	1.229 ± 0.167	1.223 ± 0.155
Tournis	40	29	0.940 ± 0.100	0.900 ± 0.100	0.970 ± 0.100	0.930 ± 0.100	^*^1.200 ± 0.100	1.10 ± 0.100

Note: Femoral neck bone mineral density (FN-BMD), total hip bone mineral density (TH-BMD), lumbar spine bone mineral density (L-BMD). The data represent the mean ± SD. ^**^P < .01 or ^*^P < .05 vs control group.

BMD = bone mineral density, FN-BMD = femoral bone mineral density, L-BMD = lumbar spine bone mineral density, TH-BMD = total hip bone mineral density, TSH = thyroid stimulating endocrine.

### 4.4. BMD of TSH-suppression therapy group and control group in postmenopausal women (Table 5, Fig. 4)

**Figure 4.**
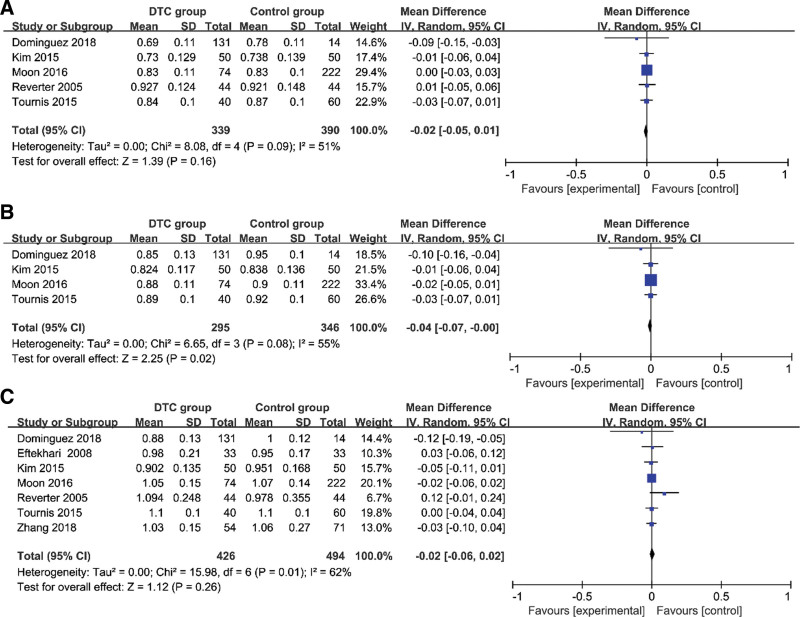
Forest plots of BMD of TSH-suppression therapy group and control group in postmenopausal women. (A) L-BMD; (B) TH-BMD; (C) FN-BMD. BMD = bone mineral density, FN-BMD = femoral bone mineral density, L-BMD = lumbar spine bone mineral density, TH-BMD = total hip bone mineral density, TSH = thyroid stimulating endocrine.

Using the inverse variance method, a random-effects model and input continuous data were determined. It showed that no significant difference existed between L-BMD and DTC patients with TSH-suppression therapy who were postmenopausal women (*I*^2^ = 51%, MD=–0.02, 95%CI=–0.05–0.10, *P* = .16) (Fig. 4A). In addition, it was found that TH-BMD was closely related to DTC patients with TSH-suppression therapy who were postmenopausal women (*I*^2^ = 55%, MD=–0.04, 95%CI=–0.07–0.00, *P* = .02) (Fig. 4B). It also indicated that there was no relationship between FN-BMD and DTC patients with TSH-suppression therapy who were postmenopausal women (*I*^2^ = 62%, MD=–0.02, 95%CI=–0.06–0.02, *P* = .26) (Fig. 4C).

**Table 5:** BMD of TSH-suppression therapy group and control group in postmenopausal women.

References	Patients	Controls	FN-BMD (g/cm^2^)		TH-BMD (g/cm^2^)		L-BMD (g/cm^2^)	
			Patients	Controls	Patients	Controls	Patients	Controls
Dominguez	131	14	0.69 ± 0.11	0.78 ± 0.11	^*^0.85 ± 0.13	0.95 ± 0.10	0.88 ± 0.13	1.00 ± 0.12
Eftekhari	33	33	N. A	N. A	N. A	N. A	0.980 ± 0.210	0.950 ± 0.170
Kim	50	50	0.730 ± 0.129	0.738 ± 0.139	0.824 ± 0.117	0.838 ± 0.136	0.902 ± 0.135	0.951 ± 0.168
Moon	74	222	0.830 ± 0.110	0.830 ± 0.100	0.880 ± 0.110	0.900 ± 0.110	1.050 ± 0.150	1.070 ± 0.140
Reverter	44	44	0.927 ± 0.124	0.921 ± 0.148	N. A	N. A	1.094 ± 0.248	0.978 ± 0.355
Tournis	40	60	0.840 ± 0.100	0.870 ± 0.100	0.890 ± 0.100	0.920 ± 0.100	1.100 ± 0.100	1.100 ± 0.100
Zhang	54	71	N. A	N. A	N. A	N. A	1.03 ± 0.15	1.06 ± 0.27

Note: Femoral neck bone mineral density (FN-BMD), total hip bone mineral density (TH-BMD), lumbar spine bone mineral density (L-BMD). The data represent the mean ± SD. ^**^P < .01 or ^*^P < .05 vs control group.

BMD = bone mineral density, FN-BMD = femoral bone mineral density, L-BMD = lumbar spine bone mineral density, TH-BMD = total hip bone mineral density, TSH = thyroid stimulating endocrine.

### 4.5. BMD of TSH-suppression therapy group and control group in men (Table 6, Fig. 5)

**Figure 5.**
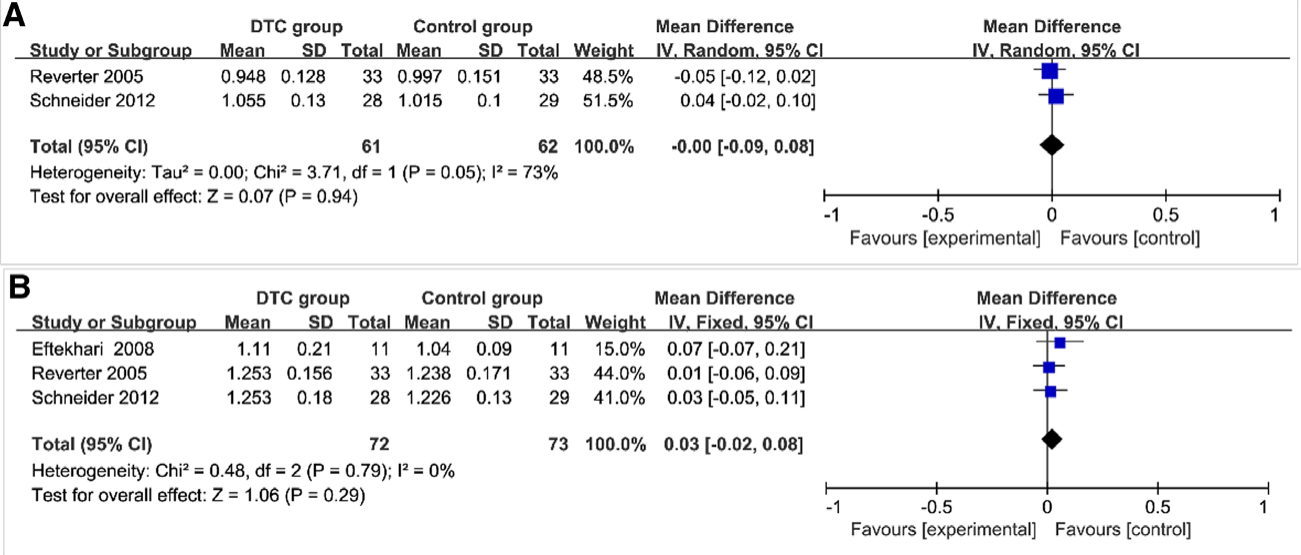
Forest plots of BMD of TSH-suppression therapy group and control group in men. (A) FN-BMD; (B) L-BMD. BMD = bone mineral density, FN-BMD = femoral bone mineral density, L-BMD = lumbar spine bone mineral density, TSH = thyroid stimulating endocrine.

Using the inverse variance method, a random-effects model or fixed-effects model and input continuous data were chosen respectively. It was found that no significant difference existed between FN-BMD and DTC patients with TSH-suppression therapy who were men (*I*^2^ = 73%, MD=–0.00, 95%CI=–0.09–0.08, *P* = .94) (Fig. 5A). It was also found that L-BMD is not related to DTC patients with TSH-suppression therapy who were men (*I*^2^ = 0%, MD = 0.03, 95%CI=–0.02–0.08, *P* = .29) (Fig. 5B).

**Table 6:** BMD of TSH-suppression therapy group and control group in men.

References	Patients	Controls	FN-BMD (gr/cm^2^)		TH-BMD (gr/cm^2^)		L-BMD (gr/cm^2^)	
			Patients	Controls	Patients	Controls	Patients	Controls
Eftekhari	11	11	N. A	N. A	N. A	N. A	1.110 ± 0.210	1.040 ± 0.090
Reverter	33	33	0.948 ± 0.128	0.997 ± 0.151	N. A	N. A	1.253 ± 0.156	1.238 ± 0.171
Schneider	28	29	1.055 ± 0.13	1.015 ± 0.10	1.099 ± 0.14	1.085 ± 0.10	1.253 ± 0.18	1.226 ± 0.13

Note: Femoral neck bone mineral density (FN-BMD), total hip bone mineral density (TH-BMD), lumbar spine bone mineral density (L-BMD). The data represent the mean ± SD. ^**^P < .01 or ^*^P < .05 vs control group.

BMD = bone mineral density, FN-BMD = femoral bone mineral density, L-BMD = lumbar spine bone mineral density, TH-BMD = total hip bone mineral density, TSH = thyroid stimulating endocrine.

### 4.6. Publication bias and sensitivity analysis

**Figure 6.**
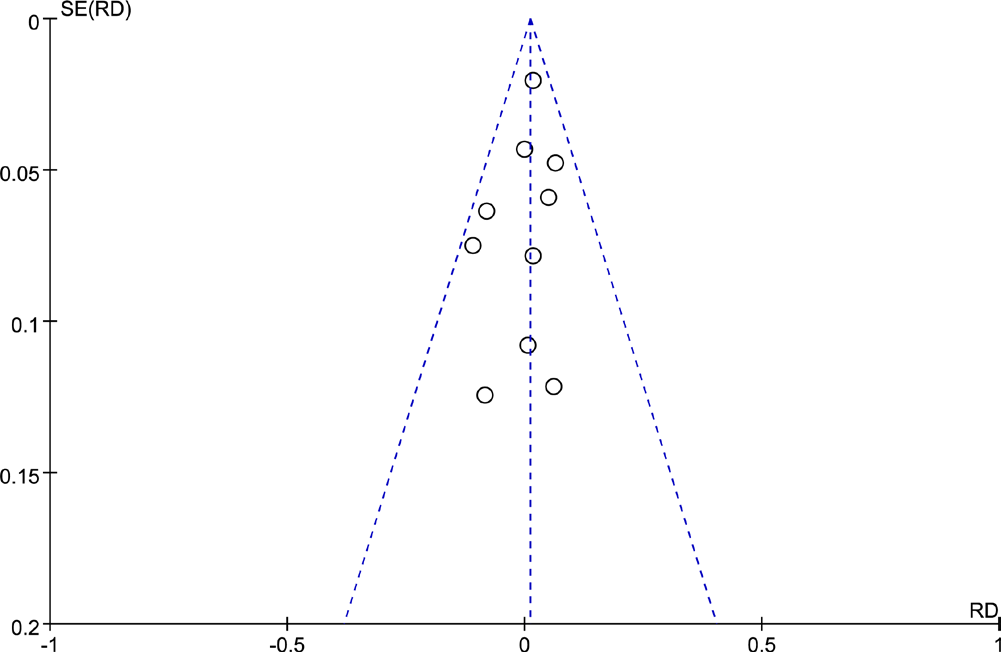
Begg’s funnel plot for publication bias analysis of the included articles.

The publication bias was evaluated by funnel plot visual inspection and Begg’s test, and no obvious asymmetric distribution was found (Fig. 6), indicating no publication bias existed.

## 5. Discussion

LT_4_ is usually used for TSH suppression therapy after DTC surgery, which makes TSH levels low and even undetectable.^[10]^ It was reported that the low level (<0.1 mU/L) of TSH may inhibit the growing of tumor cells, reducing the recurrence rate and mortality rate of DTC.^[11]^ However, it has also been revealed that the high level of TSH (> 2 mU/L) is beneficial to the fragile or older patients with DTC. Minority DTC patients need this TSH inhibition level.^[12]^ Nowadays, the concept of TSH inhibitory therapy has changed and shifted, and there is no consensus on clinical guidelines for TSH inhibitory therapy and its degree of inhibition. Both the American Thyroid Association and the European Thyroid Association conducted risk stratification for postoperative recurrence of DTC. The stratification strategy is mainly based on the primary tumor, lymph nodes, distant metastasis, whether it is residual, and type of pathological that may help reduce the tumor recurrence. Chinese Thyroid Association mainly emphasizes dual-risk assessment, which takes into account the patient’s tumor recurrence risk classification and the risk of TSH inhibitory treatment adverse reactions.

According to the recommendations of American thyroid association guidelines in 2015, the TSH level of patients with a high risk of thyroid cancer should be controlled below 0.1 mU/L. Low-risk patients are recommended to control TSH slightly below the lower limit of the normal reference range (0.1–0.5 mU/L) or maintain the lower limit of the normal reference range (0.5–2.0 mU/L). After passing the double risk assessment, regardless of the risk of TSH suppression therapy, it is recommended to always control the TSH of high-risk recurrence patients at < 0.1 mU/ L.^[13]^ Long term use of LT_4_ makes subclinical hyperthyroidism occur in DTC patients with potential adverse reactions such as cardiovascular disease.^[14]^ Osteoporosis is mainly manifested by the reduction of bone mass and the destruction of the fine structure of bone tissue, resulting in increased bone fragility and increased fracture risk.^[15]^ Besides the influence factors such as age, gender, calcium, vitamin D, and parathyroid function, hyperthyroidism is also a common high-risk factor for osteoporosis.^[16]^ However, there is no definite conclusion to figure out whether subclinical hyperthyroidism caused by the use of LT_4_ will cause bone loss and osteoporosis. Therefore, the purpose of this study was to determine the effect of TSH inhibitory therapy on BMD in DTC patients through literature review and meta-analysis.

It was reported that long-term use of LT_4_ and high cumulative doses of LT_4_ is significantly associated with an increased risk of osteoporosis in patients with thyroid cancer after thyroidectomy.^[17]^ In the present study, the effect of TSH-suppression therapy on BMD (FN-BMD, TH-BMD, L-BMD) with DTC patients were investigated in a total of 1651 patients. The results indicated that there was no significant effect on TH-BMD while significant effect on FN-BMD and L-BMD in DTC patients with TSH-suppression therapy in the present study. Furthermore, premenopausal women, postmenopausal women, and men were classified and researched, respectively.

The utilization of BMD as a predictor of future fracture rate has been well established.^[18]^ According to our meta-analysis, the relationship between lumbar BMD and DTC patients with TSH-suppression therapy who were premenopausal women was significant. A study revealed that premenopausal women were associated with decrements in lumbar spine BMD, which is similar to ours.^[19]^ In addition, we also found that total hip BMD was closely related to DTC patients with TSH-suppression therapy who were premenopausal women. Another study reported that the effect on TH-BMD and DTC patients with TSH-suppression therapy who were premenopausal women is significant, which is consistent with ours.^[20]^

In the present meta-analysis, it was found that TSH suppression therapy was associated with total hip BMD in postmenopausal women. Previous research has reported that the effect on TH-BMD and DTC patients with TSH-suppression therapy who were postmenopausal women is significant, which is similar with this meta-analysis.^[21]^ However, we also found that DTC patients with TSH-suppression therapy who were postmenopausal women had no association with L-BMD and FN-BMD. This may be due to the postmenopausal osteoporosis manifested by a decrease in lumbar vertebral bone density, which is mainly composed of trabecular bone.^[22]^ It was conjointly discovered that long-term LT_4_ suppressive medical care in premenopausal women with DTC doesn’t induce severe bone loss.^[23]^ In addition, for the difference between premenopausal and postmenopausal women, the most likely explanation may be that retained estrogen secretion counteracts the absorption of excess thyroid hormone before menopause.^[24]^

According to Kim et al, there is no effect of TSH-suppression therapy on BMD in men.^[25]^ On the other hand, it was also reported that the serum TSH concentration in the lower reference range may be related to low bone density in men.^[26]^ Our present meta-analysis found that there is no influence of TSH-suppression medical care on BMD in men, which is consistent with Kim et al’s research. In addition, Yoon et al’s similar research indicated that TSH suppression therapy is associated with a decrease in total BMD of the hip and spine in postmenopausal DTC patients, while it was not associated with both premenopausal women and men.^[27]^ This is not completely consistent with our conclusion. In our present study, it was found that TSH suppression therapy is not only related to TH-BMD but also associated with postmenopausal DTC patients.

## 6. Strengths and limitations of this study

There are still several restrictions and limitations in the current meta-analysis. Firstly, some studies have high heterogeneity. To provide a more conservative effect estimate, we tried to investigate the heterogeneity between studies using a random effects model. Secondly, diagnostic criteria cannot easily be uniform. Due to the different tools used for BMD measurement, non-standardization methods such as single or dual photon absorptiometry were used in a few studies, but not dual energy X-ray absorptiometry to diagnose BMD. Thirdly, only 10 suitable studies were included for investigating the effect of TSH-suppression therapy on BMD with DTC patients. In addition, based on our previous research’s methods,^[28,29]^ large-scale and long-term randomized controlled trials in different populations should be conducted to provide more significant evidence in future studies.

## 7. Conclusion

In the present study, the effect of TSH-suppression therapy on BMD (FN-BMD, TH-BMD, L-BMD) with DTC patients was evaluated in a total of 10 research studies. The results showed that there were some differences in the effects of TSH inhibition therapy on BMD in different parts of patients with DTC: There was no significant effect on TH-BMD in DTC patients with TSH-suppression therapy; There was significant effect on FN-BMD/L-BMD and DTC patients with TSH-suppression therapy; There was significant effect on TH-BMD/L-BMD and DTC patients with TSH-suppression therapy who were premenopausal women; There was no significant effect on FN-BMD and DTC patients with TSH-suppression therapy who were premenopausal women; There was significant effect on TH-BMD and DTC patients with TSH-suppression therapy who were postmenopausal women; There was no significant effect on L-BMD/FN-BMD and DTC patients with TSH-suppression therapy who were postmenopausal women; There was no significant effect on L-BMD/FN-BMD and DTC patients with TSH-suppression therapy who were men.

## Acknowledgments

Jingxin Mao and Yanyan Zhang conceived and designed the research. Yang Zou, Bin Li, and Jingxin Mao conducted the statistical analysis and wrote the paper. Yang Zou and Xiaodong Wang abstracted the total data from the included articles. All authors contributed to manuscript revision and read and approved the final manuscript. We also want to thank Prof Wang and Dr Sunil K. Vimal of Southwest University for kindly helping with English editing.

## Author contributions

**Conceptualization:** Jingxin Mao, Yanyan Zhang.

**Data curation:** Yang Zou, Bin Li.

**Formal analysis:** Yang Zou.

**Funding acquisition:** Bin Li, Jingxin Mao.

**Investigation:** Bin Li.

**Methodology:** Yang Zou, Xiaodong Wang, Jingxin Mao, Yanyan Zhang.

**Project administration:** Xiaodong Wang.

**Resources:** Xiaodong Wang.

**Software:** Yang Zou, Xiaodong Wang.

**Supervision:** Xiaodong Wang.

**Validation:** Bin Li, Xiaodong Wang, Jingxin Mao.

**Visualization:** Xiaodong Wang.

**Writing – original draft:** Yang Zou, Bin Li, Jingxin Mao.

**Writing – review & editing:** Jingxin Mao, Yanyan Zhang.
